# Analysis of the National Adult Nutrition Survey (Ireland) and the Food4Me Nutrition Survey Databases to Explore the Development of Food Labelling Portion Sizes for the European Union

**DOI:** 10.3390/nu11010006

**Published:** 2018-12-20

**Authors:** Michael J. Gibney, Aifric O’Sullivan, Albert Flynn, Janette Walton, Hannelore Daniel, Yannis Manios, Alfredo Martinez, Wim H. M. Saris, Eileen R. Gibney, Irina Uzhova

**Affiliations:** 1 Institute of Food & Health, University College Dublin, D04V1W8 Dublin, Ireland; aifric.osullivan@ucd.ie (A.O.); eileen.gibney@ucd.ie (E.R.G.); irina.clinton@outlook.com (I.U.); 2 School of Food and Nutritional Sciences, University College Cork, T12Y337 Cork, Ireland; a.flynn@uccd.ie (A.F.); janette.walton@ucc.ie (J.W.); 3 Molecular Nutrition Unit, Center of Life and Food Sciences Weihenstephan, Technische Universität München, D-85354 Freising, Germany; hannelore.daniel@tum.de; 4 Department of Nutrition and Dietetics, Harokopio University, 17671 Athens, Greece; manios@hua.gr; 5 Department of Nutrition, Food Science and Physiology, Center for Nutrition Research, University of Navarra, 31009 Pamplona, Spain; jalfmtz@unav.es; 6 Department of Human Biology, NUTRIM, School for Nutrition and Translational Research in Metabolism, Maastricht University Medical Centre, 6200MD Maastricht, The Netherlands; W.Saris@maastrichtuniversity.nl; 7 Department of Health and Nutritional Sciences, Institute of Technology Sligo, F92YW50 Sligo, Ireland

**Keywords:** food labelling, portion size, RACC, NANS, Food4Me

## Abstract

The present study set out to explore the option of developing food portion size for nutritional labelling purposes using two European Union (EU) dietary surveys. The surveys were selected as they differed in (a) methodologies (food diary versus food frequency questionnaire), (b) populations (Irish National Adult Nutrition Survey (NANS) versus a seven-country survey based on the pan EU study Food4Me), (c) food quantification (multiple options versus solely photographic album) and (d) duration (4 consecutive days versus recent month). Using data from these studies, portion size was determined for 15 test foods, where portion size was defined as the median intake of a target food when consumed. The median values of the portion sizes derived from both the NANS and Food4Me surveys were correlated (*r* = 0.823; *p* < 0.00) and the mean of the two survey data sets were compared to US values from the Recognized as Customarily Consumed (RACC) database. There was very strong agreement across all food categories between the averaged EU and the US portion size (*r* = 0.947; *p* < 0.00). It is concluded that notwithstanding the variety of approaches used for dietary survey data in the EU, the present data supports using a standardized approach to food portion size quantification for food labelling in the EU.

## 1. Introduction

Nutrition labelling was first introduced in the late 1990s as an attempt to guide consumers in choosing combinations of foods that meet balanced dietary guidelines, an approach which is strongly advocated by the World Health Organisation [1]. Since first introduced, nutrition labels have evolved both in the mode of presentation, with front of pack labelling incorporating Guideline Daily Amounts and involving colour-coded symbols generally referred to as the traffic light system [2,3]. A central feature of nutrition labelling is the reference weight or equivalent used on the label. A review of existing international practices shows that for most regions, data on the nutrition content of a packaged food can be expressed as serving size (in g or mL) which is generally defined as the amount estimated to be consumed at a given eating occasion, while the EU and Australia/New Zealand are unique in the use of 100 g/mL as the basis for nutrition label data [4]. Many studies have examined consumer preferences for either serving size or units per 100 g/mL in their interpretation of nutrition labelling of foods. Data from Ireland and a European industry survey clearly shows a strong consumer preference for nutrient data on food labels to be presented as portion size rather than per 100 g/mL [5,6]. However, research from the UK suggests that a standardised approach to labelling is critical, as consumers were confused when one manufacturer presented portion size data in quantitative terms while others, for the same product type, used reference values such as ‘half a pack’ [7]. In this researcher-led retail outlet survey, consumers who felt they would consume more than half a pack struggled to quantify the nutrient intake data. There was also a general feeling that portion size data could be unrealistic. This latter criticism has also come from EU consumer groups (Bureau Europeén des Unions de Consommateurs; BEUC) who argue that “*food companies can set their own serving sizes on food labels. Yet many portion sizes are unrepresentative of what people actually eat*” [8]. Research from Australia has shown significant differences between industry-derived food label portion sizes within food categories [9]. UK data also shows that serving size suggestions differ across industry, non-governmental organisations (NGOs) and health care professionals (HCPs) literature [10]. Furthermore, portion size (amount of food consumed per eating occasion) and serving size (recommended by public health nutrition agencies) can be confused [11]. 

The BEUC report on portion “urges the Commission to develop guidance on portion sizes to make sure the information provided to consumers is trustworthy and enables them to make healthful choices” [8]. In its most recent legislation, the European Commission (EC) makes a clear case for the development of portion size data for the EU: “In order to ensure the uniform implementation of the expression of the nutrition declaration per portion or per unit of consumption and to provide for a uniform basis of comparison for the consumer, the Commission shall, taking into account actual consumption behaviour of consumers as well as dietary recommendations, adopt, by means of implementing acts, rules on the expression per portion or per consumption unit for specific categories of foods” [12].

The present study set out to examine the use of food consumption databases for the development of food portion sizes for nutrition labelling in the EU. Taking into account the wide variability in the methodologies used across the EU in national and pan-EU dietary surveys, the study focused on the use of two such surveys which differed in sample selection, geographic location, study duration, survey tool and food intake quantification in order to generate portion size data for comparisons between these two disparate surveys and also with other data sets on portion size from the US. A previous study using the Food4Me database has shown that differences across the EU in mean daily intake of foods are due to differences in the frequency of consumption of foods rather than the serving size at each eating occasion [13]. Thus, portion sizes are quite similar across the EU and largely determined by gastronomic factors. A serving of pasta or a fillet of salmon is likely to be similar each time such a food is eaten in almost all geographic regions. What makes populations differ is the cultural tendency to eat specific foods with higher or lower frequencies of intake. 

## 2. Materials and Methods

Two data sets on food consumption were used to generate data on portion size for the present study. Because of their significant differences in design, the use of these two surveys present both opportunities and challenges. If these two highly contrasting studies show differences in estimates of portion size, then the challenges to policy makers would be significant in comparing the greater validity of either one of the methods. On the other hand, if these studies of quite disparate design show concordance, then the varied existing national dietary surveys across the EU can be incorporated into the decision-making process. 

### 2.1. Food4Me

The Food4Me study is a multi-centre, web-based, proof-of-principle study of personalised nutrition. The Food4Me study design and measurement methods have been previously described [14]. The aim of the study was to determine whether providing more personalised dietary advice led to greater health outcomes compared to conventional population-based advice. Participants were recruited via the Internet and other approaches, including radio, newspapers and posters. The study centres included the following: University College Dublin, Ireland; Maastricht University, the Netherlands; University of Navarra, Spain; Harokopio University, Greece; University of Reading, UK; National Food and Nutrition Institute, Poland; and Technische Universitat Munchen, Germany. Participants aged >18 years of age were recruited into the study and the cohort was as representative of the adult population as possible. 

Habitual dietary intake was quantified throughout the study at months 0, 1, 2, 3 and 6 months using an online food frequency questionnaire (FFQ) developed for the study. The Food4Me FFQ consisted of 157 food items. The well-validated EPIC-Norfolk FFQ (version CAMB/PQ/6/1205) [15] was used as a guide for food items and food group categories. In developing the Food4Me FFQ, the original 130 food items presented in the EPIC-Norfolk FFQ were expanded upon to incorporate an additional 27 commonly consumed food items that were considered nutritionally important across the seven EU countries in the Food4Me study. For all food items, frequency of intake was estimated by asking, “How often would you have consumed each of the following in the past month?” and participants could select their frequency from nine categories of intake ranging from “never (<1 per month)” to “6+ per day”. After selecting their frequency of consumption, participants were asked to choose their usual serving size from a range of portion size pictures for each food item. The choice of portion sizes available to subjects for selection was calculated by selecting food codes from the NANS database and matching these with each of the foods listed in the FFQ. These were then merged and recoded into a single food code for each food listed in the Food4Me FFQ and the 25th, 50th and 75th percentiles of eating occasion intake which represent small, medium and large portion sizes. Options for portion sizes above, below and in between these percentiles were also provided, resulting in seven portion choices. The online Food4Me FFQ was pre-programmed to ensure that a frequency of consumption was reported for every food item before the participant could submit the FFQ and was designed so that participants could check and/or modify previous responses before submitting the FFQ. Intakes of foods and nutrients were computed in real time using a food composition database based on McCance and Widdowson’s *The Composition of Foods* [16,17]. The development and validation of the Food4Me online FFQ is described elsewhere [18,19]. For the purpose of the analysis in this paper, only baseline (month 0) intake data were used. 

### 2.2. National Adult Nutrition Survey (NANS; Ireland)

The fieldwork phase of NANS was carried out between October 2008 and April 2010 by the Irish Universities Nutrition Alliance (IUNA, http://www.iuna.net). A more detailed description of the survey methodology has been reported previously [20]. Eligible participants were aged 18 years and over who were free-living and who were not pregnant or breast feeding. Adults were randomly selected from a database of names and addresses held by Data Ireland (Irish postal service). Random selection was designed to provide a sample representative of urban and rural areas and deliver at least 100 individuals in the least-populated age and sex subgroups. In total, 1500 adults aged 18–90 years (740 men and 760 women) took part in the dietary survey. The sample was representative of the Irish population with respect to gender, age, location, and social class as per the 2006 Irish census (Central Statistics Office 2007).

Food and beverage intake was determined using a 4-day semi-weighed food diary. Respondents were asked to record detailed information regarding the amount and types of all foods, beverages and nutritional supplements consumed over the recording period and where applicable, the cooking methods used, brand names of the foods consumed and details of recipes. The data on a total number of 2552 food items was collected. Food and beverage intake was assessed using WISP V3.0 (Tinuviel Software, Anglesey, UK), based on data from the McCance and Widdowson’s *The Composition of Foods* (5th and 6th editions) [16,17] supplemented with Irish food codes, to calculate nutrient intakes. In addition, the survey identified the approach used to quantify the intake of each food from seven possible choices: food photographic atlas, weighed intake by the participant, manufacturer’s portion sizes, official published portion estimates, household measures, estimated values by the researcher and typical weights determined by the researcher based on data derived from previous surveys. 

### 2.3. Food Category Choices and Calculation of Portion Size Data

In consultation with industry nutritionists from the funding companies, the following test foods were proposed to provide as wide a challenge to portion size estimation as possible: cheese, chocolate, cakes, ice cream and sorbet, margarine, mayonnaise, ready-to-eat breakfast cereals (RTEBC), savoury snacks, sweet biscuits, sweet bread toppings and spreads (jams, marmalade, nut-based spreads) and sugar confectionery. These food categories were broadly representative of published US data for portion size using the tables for reference amounts customarily consumed (RACC) [21]. 

As with other countries, such as the US and Australia [9,21], the median amount of food consumed during an eating occasion on a population basis was the basis for calculating average portion size. When estimates of a mean intake of a food include non-consumers, as frequently occurs in research papers, the corresponding median intake is frequently zero, simply because large numbers of non-consumer values are zeros themselves. For that reason, non-consumers are always excluded, and median intakes are calculated to determine portion size. 

Two approaches are possible in the use of food intake data to estimate median portion size per eating occasion where multiple opportunities exist for each consumer to select a portion size. This can apply to the NANS database but not the Food4Me database where the FFQ allows only one choice in serving size. The first approach is to pool all eating occasions into a single distribution to calculate the median value. Thus, the number of points on the distribution will exceed the number of consumers. Another approach would be to establish a mean value across eating occasions for each consumer and then to use the distribution of these mean values for median intake estimation. In this approach, the number of points on the distribution equals the number of consumers. The former was referred to as the population distribution with the latter as the individual distribution. 

All of the data in the NANS and Food4Me studies were contained in databases using SPSS software (SPSS Statistics version 20; SPSS Inc., Chicago, IL, USA) and the present study analysis used this software. The agreement between the portion sizes derived from Food4Me, NANS and established values for RACC was estimated using Pearson correlation coefficient.

## 3. Results

Table 1 describes the basic characteristics of the two studies. Appropriate statistical power calculations were conducted in each study to ensure that the sample size was adequate for the research objectives of both studies, which included measurements of food intake. Whilst the studies differed in their recruitment process, with NANS recruiting to be nationally representative and Food4Me recruiting more generally from the adult population, both studies showed similarities across the populations. In line with prevalence across Europe, just under half (44.8%) of Food4Me participants had a BMI 25.0 kg m^2^ and 32.1% were physically inactive individuals. The sample in the Food4Me study was broadly representative of the demography in each country as measured by the age range of participants, gender distribution and health concerns. In the NANS study, seven different options to quantify food intake were available for use by the researcher in discussion with the participant—as is the nature of food records, where multiple ways exist to quantify intakes. In the case of the Food4Me study, the only option available for quantifying food intake was the photographic atlas, which presented all seven estimates of portion size for each food. In the case of NANS, the most widely used tool to determine the weight of a food serving was direct weighing by the participant. Manufacturers’ portion size data was among the least used for quantification (Table 1). The Food4Me study had a higher proportion of females, a higher reported energy intake and a lower prevalence of overweight and obese. 

The two options that were available to estimate portion size using the NANS data (individual versus population-based) showed a high degree of correlation (*r* = 0.996; *p* < 0.00). Given that the Food4Me data can use only individual-based distributions of amounts consumed per eating occasion for estimating median portion size, all subsequent use of NANS data uses the individual-based approach to creating such distributions. Table S1 (see Supplementary Materials) gives full statistical profiles of both approaches to estimating median intake per eating occasion. 

The estimates of portion size of the selected foods from both the NANS and the Food4 Me study are given in Table 2. The data show a high degree of correlation (*r* = 0.823; *p* < 0.00). However, there are several food categories where each method yields slightly different data. This is due to the challenge of re-aligning food codes into different food categories. In NANS, a total of 2319 food codes were re-aligned into 71 food categories. In Food4Me, the FFQ focused on 157 foods or food groups. Whereas it was possible to re-code all the NANS data into codes directly corresponding to the categories used in the Food4Me database, for the present study, the use of the existing food category data for each database helps illustrate the challenges of choosing categories for food portion estimation for nutrition labelling purposes. 

**Figure 1:**
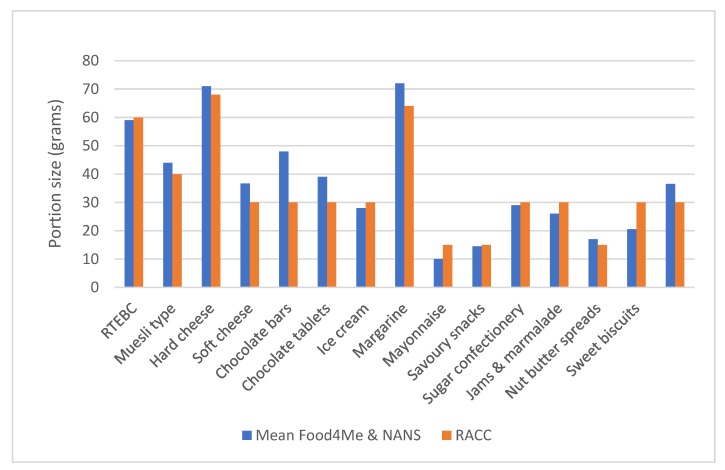
A comparison of values for portion size (g) derived as the mean of median intakes from Food4Me and NANS studies and those from the US Recognised as Customarily Consumed (RACC) database for the 15 food categories chosen in the study.

In order to get an international comparison, the median portion sizes from the two studies (NANS and Food4Me) were averaged and compared with US data from the US Recognised as Customarily Consumed (RACC) database. These are shown in Figure 1 and the two sets of values are highly correlated (*r* = 0.947; *p* < 0.00). Individually, the two data sets were also well correlated with the RACC data for the US (NANS: *r* = 0.964; *p* < 0.00 and Food4Me: *r* = 0.844; *p* < 0.00). 

## 4. Discussion

As with any study, certain weaknesses exist and these merit discussions. To begin with, the analysis could be considered biased in that the NANS database draws on 1500 Irish adults while the Food4Me database of 1269 subjects also included 170 subjects from Ireland. However, the two sets of Irish subjects were recruited differently and had their food intake measured with two markedly contrasting methodologies, thus reducing the likelihood of a major bias in the interpretation of the data. A second limitation is that in the present study we used 15 food categories to draw our conclusions and thus these conclusions can only be definitive for these categories. However, the data indicate a high level of agreement in the estimate of portion size of these foods, which suggests the likelihood that if a larger number were chosen, a broadly similar conclusion would be drawn. Nutrition surveys entail several thousand foods organized into a smaller number of categories, typically between 50 and 150 categories. However, for the purposes of regulating portion sizes, a much smaller number of categories can be anticipated. Thus, the US system of reference amounts commonly consumed lists only 21 broad food categories and within these just 143 sub-categories. In any EU analysis the number of food categories and sub-categories would be of a similar size and well below the total number of individual foods recorded within the survey. 

Although existing EU legislation permits the use of both nutrients per 100 g/mL or nutrients per portion size, there is no legislative base for determining the latter. This has led to consumer groups to call for the European Commission “*to develop guidance on portion sizes to make sure the information provided to consumers is trustworthy and enables them to make healthful choices*” [8] The Commission itself has expressed its willingness to pursue the development of food portion sizes for nutrition labelling noting that it will take into account “*actual consumption behaviour of consumers as well as dietary recommendation*” [12]. Unlike the United States, which uses a single national dietary survey (The National Health and Nutrition Evaluation Survey; NHANES) [22], each member state of the European Union conducts its own national nutrition survey, each of which has been documented in detail by The European Food Safety Authority (EFSA) [23,24]. These national surveys differ in many aspects: sample size and recruitment, dietary assessment tool, food quantification approach, survey duration, food coding systems and so forth. The present study selected for use two surveys that differ in each of these parameters. Nonetheless, the results of the present study show that estimates of food portion size in both surveys, based on the median intake of a given food among all eating occasions among consumers of the food, showed remarkable similarity for 15 widely different test foods. Thus, the clear message from this study is that the great diversity of dietary surveys across the EU should not be a barrier to the development of valid portion size estimates for use in nutritional labelling systems. In the EU, food intake data range from those with a single value per subject for portion size for a given food, such as with the use of food frequency questionnaires, to multiple portion sizes for each individual, where several days are used and where multiple options for estimating food weights are available. The present study explored this in terms of estimating median portion size with individual only data or population data, combining multiple records of portion size for all consumers. Given the similarity of estimated portion size using either method, a default should be the use of individual-based data for the estimation of portion size. 

Nonetheless, a number of challenges would need to be faced to provide a comprehensive set of data on potion size. Data collected on food intake at the individual level can be as detailed as possible, right down to brand level. For nutritional analysis, food intake data must be re-aligned to the definitions of foods in the food composition tables in use. Food chemical exposure studies also require this re-alignment of food codes into food categories in which a particular food chemical is approved for use. Extensive guidelines on such have been included in the EFSA database covering food categorisation for estimates of exposure to additives, packaging materials, pesticides and contaminants [24]. If the EU is to proceed with the development of reference values for portion size in nutrition labelling, a similar set of guidelines will have to be developed. In the US, the RACC system lists 21 broad food categories, each of which is expanded into specific food types within each category [21]. Thus, the broad category ‘Dairy products’ includes the following sub-categories for cheese: cottage cheese; cheese used primarily as ingredients, e.g., dry cottage cheese, ricotta cheese; cheese, grated hard, e.g., Parmesan, Romano; cheese. This definition would see traditional European cheeses such as Gouda, Brie, Leicester and Camembert all classified together with a common portion size. Defining separate portion sizes for each specific European cheese is only possible if the intake of that cheese was recorded as such in a national dietary survey. Moreover, the number of consumers of that individual cheese would have to exceed some cut-off point above which adequate data would exist to allow for an accurate assessment of population median intake at such cheese eating occasions. Thus, defining food categories for use in any EU reference database on portion sizes commonly consumed will be a significant challenge. Indeed, an ab initio survey that used a simple harmonized photographic tool applied to representative samples in all member states might provide better data than could be extracted from existing databases. In the area of food regulatory affairs, the definitions of foods often do not correspond with those used by nutritionists (‘chocolate tablets’ representing chocolate bars that can be broken into squares or muesli, a generic term as opposed to Cruesli, a brand term.

Beyond these technical challenges of food classification lies another major issue which pertains to foods sold in packages, where the package is intended to provide multiple portions. In the US, the basic RACC values are adjusted, depending on the nature of the packaging arrangement [25]. For example, for products in discrete units (e.g., muffins, sliced products, such as sliced bread, or individually packaged products within a multi-serving package), the exact use of the RACC value depends on the base product relative to the RACC value. Thus, the US regulation states: “*If a unit weighs 50 percent or less of the reference amount, the serving size shall be the number of whole units that most closely approximates the reference amount for the product category*” [21]. Thus, a slice of bread might weigh 25 g, for example, but the median intake at a bread eating occasion might be 50 g, in which case it is assumed that two units make up a portion size. The regulations proceed with further unit values relative to the reference amount: <50%, >0 <67%, >67 <200% and 200% or greater, each with its own unique qualifying statements. The definitions continue for other categories: products in large discrete units that are usually divided for consumption (e.g., pizza), non-discrete bulk products (e.g., breakfast cereal), products which consist of two or more foods packaged and presented to be consumed together where the ingredient represented as the main ingredient is a bulk product (e.g., peanut butter and jelly) and many other sub-categories of food types. A broadly similar approach has also been adopted in Canada [26]. Thus, establishing an estimate of the median of a food consumed among eating occasions of that food is effectively a risk assessment stage. The translation of that data into nutritional labelling value is a risk management strategy which must involve a wide range of stakeholders.

A final issue that will inevitably arise is the conflict between estimates of portion sizes commonly consumed and serving sizes recommended for optimal nutritional wellbeing. Thus, it is well recognised that portion sizes of common foods have increased considerably over the last several decades and this includes not just packaged foods but also portion sizes of home prepared foods [27]. The question will inevitably arise as to whether the food portion size used in nutrition labelling should reflect the median intake among consumers or, where such intakes are deemed to have become excessive, a proposed serving size more in line with optimal food servings to meet public health nutrition food-based dietary guidelines. Although portion sizes have increased over time, a problem still exists in the under-reporting of food energy in dietary surveys. However, one study has examined portion size estimates among subjects with either an acceptable level of energy intake or a putative under-reporting of energy intake based on conventional cut-off levels. Portion sizes were the same in under-reporters of energy intake as in accurate reporters for both ‘healthy’ and ‘less healthy’ foods. Thus, energy under-reporting may relate more to ‘omission’ of foods rather than underestimating portion weight [28]. All things considered, the establishment of accurate and independently derived values for portion sizes generally consumed is an area which needs to be addressed within the EU regulatory framework. Public health policy must choose between actual and desirable common portion sizes and must conduct research into consumer attitudes on how best to present such data on food packages. 

## 5. Conclusions

Notwithstanding these issues, the results of the present study clearly show that, taking into account the great variety of approaches to completing national food surveys across the EU, it should still be possible to use such data to determine portion sizes for use in food package nutrition labels.

## Figures and Tables

**Table 1:** Subject and survey characteristics of Food4Me and Irish National Adult Nutrition Survey (NANS) databases. IUNA: Irish Universities Nutrition Alliance.

	Food4Me	NANS
**Subject Characteristics**		
Number of countries	7	1
Subject (*n*) (% males)	1607 (39.1)	1500 (49)
Mean age (years)	39.8 ± 13.1	39.5 ± 13.1
Body Mass Index (%)		
Normal weight	53.8	34.4
Overweight	30.2	39.3
Obese	14.6	26.3
Macronutrient intakes		
Energy (MJ/day)	10.3 ± 2.9	8.6 ± 2.8
Carbohydrate (% daily energy)	46.8 ± 7.6	42.6 ± 6.9
Fat (% daily energy)	35.4 ± 6.2	34.8 ± 6.3
Protein (% daily energy)	17.1 ± 3.4	16.9 ± 3.8
**Survey Information**		
**Dietary Survey Food Record Type**	**Food Frequency Questionnaire**	**4-Day Semi-Weighed Diary**
Quantification method (%)		
Weighed by participant	0	47.2
Manufacturer’s weights	0	9.9
Photographic food atlas/Photographic portion size	100	15.6
IUNA weights	0	4.0
Published food Portion Sizes	0	10.1
Household measures	0	10.6
Estimated by researcher	0	2.5

**Table 2:** Estimates of portion size from both the NANS and the Food4Me study.

Food Category	NANS Survey	Food4Me Survey
	Intake (g) per Eating Occasion per Individual	
	Median ± SD	
Muesli, Cruesli, Granola	75 ± 38	70 ± 25
Non-whole grain (puffed flakes) and whole grain (bran flakes) cereals	45 ± 27	43 ± 13
Cakes	63 ± 44	79 ± 21
Hard cheeses	33 ± 22	40 ± 13
Soft white cheeses and spreadable. cream cheeses	30 ± 32	66 ± 20
Chocolate bars	30 ± 24	48 ± 14
Chocolate tablets	25 ± 22	31 ± 15
Ice cream	72 ± 43	64 ± 23
Margarine	10 ± 8	10 ± 4
Mayonnaise	15 ± 18	14 ± 5
Savoury snacks (e.g., salty biscuits, crackers, pretzels)	28 ± 25	30 ± 9
Sugar confectionery	30 ± 32	22 ± 17
Jam, marmalade, spreads	15 ± 13	19 ± 6
Nut butter spreads (e.g., peanut or almond butter)	20 ± 18	21 ± 11
Sweet biscuits	25 ± 22	48 ± 66

## Supplementary Materials

The following are available online at http://www.mdpi.com/2072-6643/11/1/6/s1. Table S1: Statistical aspects of the estimates of food portion size using median intakes from the NANS database for the two options for computing median intakes: population-based or individual-based.
