# Supplementary material for: Analysis of the National Adult Nutrition Survey (Ireland) and the Food4Me Nutrition Survey Databases to Explore the Development of Food Labelling Portion Sizes for the European Union
Source: Nutrients. 2018 Dec 20;11(1):6. doi: 10.3390/nu11010006 (PMC6356260; doi:10.3390/nu11010006)
Supplement: Supplementary file 1 [file nutrients-11-00006-s001.pdf]

Table S1. Statistical aspects of the estimates of food portion size using median intakes from the NANS database for the two options for computing median intakes: population-based or individual-based.

	Eating occasion intakes (g/d) individual									Eating occasion intakes (g/d) population								
	Mean	Median	Mode	Min - max	SD	SEM	Percentile			Mean	Median	Mode	Min - max	SD	SEM	Percentile		
							25th	50th	75th							25th	50th	75th
RTEBC	51	45	38	9 - 230	27	0.90	33	45	63	49	41	38	6 - 288	29	0.61	30	41	60
Muesli type	74	73	100	15 - 235	38	3.15	50	73	91	73	70	87	15 - 235	38	1.98	49	70	90
Cakes	73	63	60	5 - 457	44	1.70	45	63	90	71	60	60	5 - 457	44	1.15	40	60	91
Hard cheese	37	34	20	1 - 200	22	0.74	22	34	45	37	34	20	1 - 200	25	0.57	20	34	45
Soft cheese	37	30	30	2 - 210	29	1.88	18	30	42	36	30	17	2 - 265	33	1.77	17	30	42
Chocolate tablets	32	26	45	4 - 150	22	1.92	17	26	45	32	26	26	2 – 150	22	1.62	18	26	45
Chocolate bars	37	31	50	3 - 300	25	0.96	21	31	48	33	26	25	1 - 300	24	0.60	17	26	46
Ice cream	80	72	75	10 - 390	43	2.37	53	72	95	80	70	75	10 - 600	48	2.28	51	70	96
Margarine	13	10	8	1 - 54	8	0.28	7	10	16	12	8	8	1 - 96	10	0.16	6	8	16
Mayonnaise	19	15	15	1 - 210	18	0.82	10	15	25	19	15	15	1 - 210	18	0.63	10	15	23
Savoury snacks	35	28	25	5 - 350	25	1.09	25	28	37	34	25	25	4 - 350	26	0.80	25	25	37
Sugar confectionery	36	30	37	1 - 250	32	1.79	20	30	42	33	28	37	1 - 500	35	1.40	15	28	40
Jams & marmalade	17	15	8	3 - 144	13	0.55	8	15	20	16	15	8	1 - 144	12	0.31	8	15	19
Nut butter spreads	26	20	8	5 - 80	18	2.77	12	20	36	26	20	16	4 - 120	21	2.35	12	20	36
Sweet Biscuits	30	25	26	2 - 357	22	0.73	17	25	36	26	20	14	2 - 474	22	0.42	13	20	32
